# Efficacy of ultrasound guided caudal epidural steroid injection with or without ozone in patients with lumbosacral canal stenosis; a randomized clinical controlled trial

**DOI:** 10.1186/s12891-023-06451-5

**Published:** 2023-04-29

**Authors:** Seyed Mansoor Rayegani, Vahid Soltani, Mohsen Cheraghi, Mohammad Reza Omid Zohor, Arash Babaei-Ghazani, Seyed Ahmad Raeissadat

**Affiliations:** 1 Physical Medicine and Rehabilitation Research Center, Shahid Beheshti University of Medical Sciences, Tehran, Iran; 2 Neuromusculoskeletal Research Center, Iran University of Medical Sciences, Tehran, Iran; 3 Clinical Development Research Center of Shahid Modarres Hospital, Physical Medicine and Rehabilitation Research Center, Shahid Beheshti University of Medical Sciences, Tehran, Iran

**Keywords:** Caudal epidural steroid, Ozone therapy, Lumbar spinal stenosis, Ultrasound Guidance

## Abstract

**Background:**

Lumbosacral canal stenosis is known as the most common cause of back surgery with several complications. Selecting a minimally invasive treatment with high efficacy in such patients is necessary. This study was designed to evaluate the effectiveness of ozone therapy in combination with caudal epidural steroid in patients with lumbar spinal stenosis.

**Methods:**

A double-blind randomized clinical trial was conducted on 50 patients with lumbar spinal stenosis allocated into two study groups. Under ultrasound guidance, the first group received 80 mg of triamcinolone hexavalent with 4 mL of Marcaine 0.5% and 6 mL of distilled water to the caudal epidural space. The second group received an injection similar to the first group, combined with 10 mL of ozone (O2-O3) gas at a concentration of 10 µg/cc. The patients were followed at baseline, one, and six months after injection with clinical outcomes measures using Visual Analog Scale (VAS), Walking Distance (WD) and Oswestry Disability Index (ODI).

**Results:**

The mean age of subjects, 30 males (60%) and 20 females (40%), was reported as 64.51 ± 7.19 years old. Reduction of pain intensity based on VAS score was statistically significant in both groups at follow-up periods (P < 0.001). The VAS changes in the first month and sixth months showed no significant difference between the two groups (P = 0.28 and P = 0.33, respectively). The improvement in disability index (ODI) in both types of treatment during follow-up was significant (P < 0.0001), and there was no difference between the two treatment groups in one month and six months (P = 0.48 and P = 0.88, respectively). As for walking distance, the improvement process with both types of treatment during follow-up periods was significant (P < 0.001). However, after one and six months of treatment, the rate of improvement in patients’ walking distance in the caudal epidural steroid injection plus ozone group was significantly higher than in the epidural steroid group (p = 0.026 and p = 0.017, respectively).

**Conclusions:**

In this study, the results of VAS and ODI outcomes showed that caudal epidural steroid injection combined with ozone has no advantage over caudal epidural steroid injection alone. Interestingly, our results demonstrated that the group receiving caudal epidural steroid injection plus ozone scored significantly higher on the walking distance index than the group receiving caudal epidural steroid alone.

**Trial Registration:**

IRCT IRCT20090704002117N2 (registration date: 07/08/2019).

## Introduction

Lumbar spinal stenosis cause a compressive effect on the spinal cord or nerve roots. Stenosis can occur in the central spinal canal, lateral recesses, or intervertebral foramina [1]. The spinal canal stenosis can be congenital or acquired [2]. Most cases of spinal stenosis are acquired and are secondary to degenerative changes [3]. Clinical signs of spinal stenosis include low back pain (LBP), unilateral or bilateral neurologic deficit in lower limbs and neurogenic claudication [4]. Although the incidence rate of symptomatic types of lumbosacral canal stenosis has not yet been determined, it is the most common indication for lumbar spine surgery in people over 60 years of age [5].

Canal stenosis can be distinguished from other spinal disorders by its chronic, primarily bilateral, insidious onset [6]. Central disc herniation causes pain similar to that induced by canal stenosis, but it usually starts suddenly and increases with sitting and is accompanied by a positive sciatica test and neurological findings [7]. In indistinguishable cases, electrodiagnostic tests, imaging findings, and infectious tests for rare diseases such as hydatid cysts of the spine or brucellosis and tuberculosis can be helpful in differentiating the source of pain and symptoms [8].

In many cases, spinal canal stenosis can be treated non-surgically. Spinal stenosis treatment includes rest, weight loss, physiotherapy, epidural injections, and decompression surgery if the patient is not satisfied with non-surgical treatments [9]. The analgesic property of ozone (O2-O3) injection has been used in various diseases, including myofascial pain and joint disorders [10, 11]. One of the recently proposed treatments for spinal stenosis is ozone (O2-O3) therapy as a subset of “complementary medicine”. The use of O2-O3 gas in many cases causes partial opening of the canal and reduction of symptoms, especially sciatica, in patients with spinal stenosis [12]. In this method, a medical ozone generator converts divalent oxygen to trivalent oxygen and is injected into the area of pain, inflammation and injury within 14 s. After the injection, monovalent oxygen is gradually released at the site, helping to reduce pain and repair injury; injecting steroids into the lower back reduces the severity of related pain and the need for surgery [13]. Since ozone, like steroids, blocks phospholipase, replacing steroids with ozone, which is a much safer drug and has a similar mechanism of action to steroids, would be a logical and justifiable step [14]. Ozone also increases blood circulation at the cellular level and thereby can be effective in relieving pain caused by spinal stenosis. In addition, ozone therapy by the transforaminal approach is more effective in reducing pain caused by lower lumbar disc herniation than steroid injections [14].

Decompression surgery has many complications such as recurrence, bone degeneration, failed back surgery syndrome (FBSS) and postoperative mortality. In addition, expenses for diagnostic and treatment measures, disability, long-term leave and absence from work are among the losses that the government and individuals must pay for this disease. Therefore, choosing a minimally invasive treatment with high efficacy is necessary for these patients. In line with this goal and the absence of sufficient studies in this field, this study was designed and conducted to evaluate the efficacy of ozone therapy in patients with lumbar spinal stenosis.

## Methods

This randomized, double-blind, parallel-group clinical trial in a 1:1 allocation ratio was conducted on patients with lumbar spinal stenosis requiring intervention referred to Shahid Modarres Hospital in Tehran, Iran, from September 2019 to December 2020. All patients signed written informed consent before enrollment. The Ethics Committee approved the study protocol of Shahid Beheshti University of Medical Sciences. The trial was registered on the Iranian Clinical trial Registry with identification number IRCT IRCT20090704002117N2 (registration date: 07/08/2019). Patients were able to leave the study at any time if they were reluctant to continue the trial. This study conforms to all CONSORT guidelines and reports the required information accordingly.

In simple randomization with a 1:1 allocation ratio, 50 patients were randomly divided into two groups of 25, selected using a computer-generated list of random numbers. A physical medicine and rehabilitation resident generated the allocation sequence, enrolled patients, and assigned them to interventions. Participants and assessors were blinded to type of treatment. Due to the presence of ozone gas, there was no way to blind the physician. Inclusion criteria in this study were duration of symptoms more than 1 month and less than 1 years, Visual Analog Scale (VAS) score equal to or greater than 7, age between 50 and 70 years, body mass index (BMI) between 20 and 32 kg/m2, neurogenic claudication and magnetic resonance imaging (MRI) confirmation of lumbar spinal stenosis. The presence of neurogenic claudication was mandatory for inclusion criteria. Patients with low back pain without neurogenic claudication were not included in the study. Exclusion criteria were uncontrolled diabetes mellitus, neuropathy, spondylolisthesis, extruded disc, grade 4 knee osteoarthritis, history of previous lumbar spine surgery, scoliosis, pregnancy, clinical or laboratory evidence of infection, coagulopathy, and symptoms of cauda equina syndrome. In addition, we decided to exclude patients whose symptom duration was less than 1 month since we still hoped that they could recover with other treatments. Regarding the increase in duration of symptoms to more than 12 months, even though there is no contraindication for injection, we also excluded this group from the study. It is due to the possibility of non-response to caudal injection and depriving patients of surgical treatment.

In addition, both groups were given similar therapeutic exercises for lumbosacral canal stenosis. The purpose of these exercises was to reduce extension forces on the lumbar spine. These exercises include hip flexor, hamstring, and lumbar paraspinal muscles stretching along with abdominal (pelvic tilts and trunk raise) and gluteal strengthening. Frequency, dose, and instruction of these exercises were prescribed based on Bodack’s study entitled “therapeutic exercise in the treatment of patients with lumbar spinal stenosis” [15].

### Data collection

A physical medicine and rehabilitation specialist with more than 10 years of experience in spinal procedures performed caudal injection. After reviewing the inclusion criteria, the first group was injected with 80 mg of triamcinolone hexavalent with 4 mL of marcaine 0.5% and 6 mL of distilled water during the intervention. The second group received an injection similar to the first group with the addition of 10 mL of ozone (O2-O3) gas at a concentration of 10 µg/cc. The dose of consumed O2-O3 gas was selected by reviewing previous studies in the field of transforaminal and intradiscal injections and the absence of a similar study on the use of O2 -O3 gas in caudal epidural steroid injection to ensure safety and efficacy of treatment. Patients were followed up one and six months after injection. During this period, clinical outcomes were collected using Visual Analog Scale (VAS) for pain intensity, Oswestry Disability Index (ODI), and Walking Distance (WD) [16, 17]. The primary outcome measure was VAS. VAS scores ranging from 0 (no pain) to 10 (worst experienced pain) were individually marked. Secondary outcome measures included ODI and WD.

A curvilinear transducer at 5 to 12 MHz was used as the US instrument (Mindray M7, Shenzhen Mindray Bio-Medical Electronics Co., Ltd). The patient was in the prone position. First, sacral area was prepped and the legs were rotated internally to open up injection site. The transducer was covered with a sterile sheet. Then the probe was placed in the long axis position to scan for sacral hiatus. Following local anesthetic infiltration, a 3.5-inch 22G spinal needle was inserted with an in-plane technique. After passing the sacrococcygeal ligament to confirm the location of the needle, the probe was rotated to observe the needle in sacral hiatus in short axis. Then we turned on the color Doppler. After negative blood aspiration and observing turbulence of injectate drug was completely injected.

### Statistical analysis

In the descriptive part, mean and standard deviation were determined for quantitative variables and absolute and relative frequency for qualitative variables. The groups were compared and matched in terms of contextual variables (age, gender, duration of symptoms, clinical condition, and BMI). In the inferential section, according to the type and distribution of data using Kolmogorov-Smirnov tests, appropriate statistical methods were selected in terms of parametric or non-parametric nature. Wilcoxon test was used for pairwise comparisons of patients’ conditions at different time intervals. The repeated measures analysis of variance (ANOVA) was used to compare the mean score of pain intensity, ODI, and WD over three-time points (baseline, one month, and six months after intervention). The repeated ANOVA test appears more powerful because it separates between-subject variability from within-subject variability. The GEE model was applied for analysis because the sample size was small, and the variables were non-normal. Analyzes were performed on within-group and between-group differences. Data were analyzed by SPSS version 25 software at a significant level of P˂0.05.

## Results

**Fig. 1:**
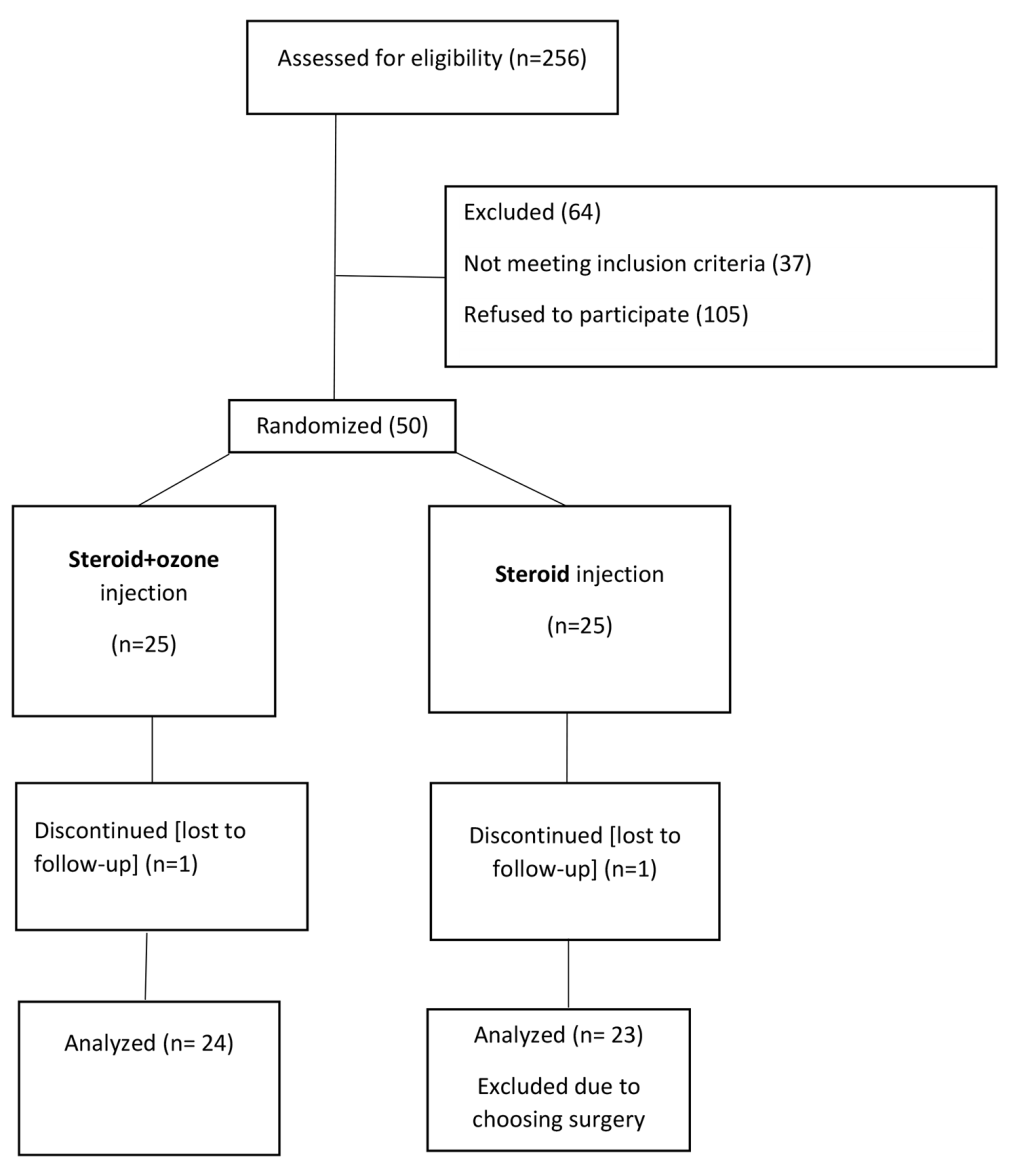
Flowchart of the study population

Out of 50 patients, 25 were in the group receiving epidural steroid injection alone and 25 in the group receiving caudal epidural steroid injection plus ozone. The group receiving epidural steroid injection alone consisted of 14 males (56%) and 11 females (44%), and the group receiving caudal epidural steroid injection + ozone consisted of 16 males (64%) and 9 females (36%); these differences were not statistically significant (p = 0.96). One participant from the steroid group and one participant from the ozone group refused to continue the study after the injection, and one patient from the steroid group also chose surgery after the injection due to the aggravation of symptoms and was excluded from the study (Fig. 1). Considering the strict entry criteria for the study, especially VAS equal to or more than 7 and cultural characteristics of the Iranian people regarding spinal interventions, known as invasive procedures, the number of people who did not agree to enter the study or did not have the conditions to enter the study was very high.

The mean age of patients in the study was 64.51 years, as 64.56 years in the group receiving epidural steroid injection alone and 64.45 years in the group receiving caudal epidural steroid injection + ozone; this difference is not statistically significant (P-value = 0.67). Other contextual and demographic variables are reported in Table 1.

**Table 1:** Demographic Characteristics of Patients

	Total n = 50	CS n = 25	CS + Ozone n = 25	P value
**Age, year**				
Mean$\pm$SD	64.51 ± 7.19	64.56 ± 7.14	64.45 ± 7.39	0.96
Median (IQR)	67 (11)	67 (11)	67 (12)	
**Sex, n(%)**				
Male	30 (60)	14 (56)	16 (64)	0.67
Female	20 (40)	11 (44)	9 (36)	
**BMI, kg/m** ^**2**^				
Mean$\pm$SD	25.25 ± 1.70	25.18 ± 1.98	25.32 ± 1.6	0.78
Median (IQR)	25.60 ± 1.91	25.60 (3.3)	25.67 ± 1.93	
**Duration of symptoms**				
Mean$\pm$SD	9.95 ± 3.30	9.06 ± 3.65	9.33 ± 3.15	0.59
Median (IQR)	9 (5)	10 (7)	9 (4)	
**Diabetes**				
Yes	6 (12.8)	3 (13.1)	3 (12.5)	0.95
No	41 (87.2)	20 (86.9)	21 (87.5)	

**Fig. 2:**
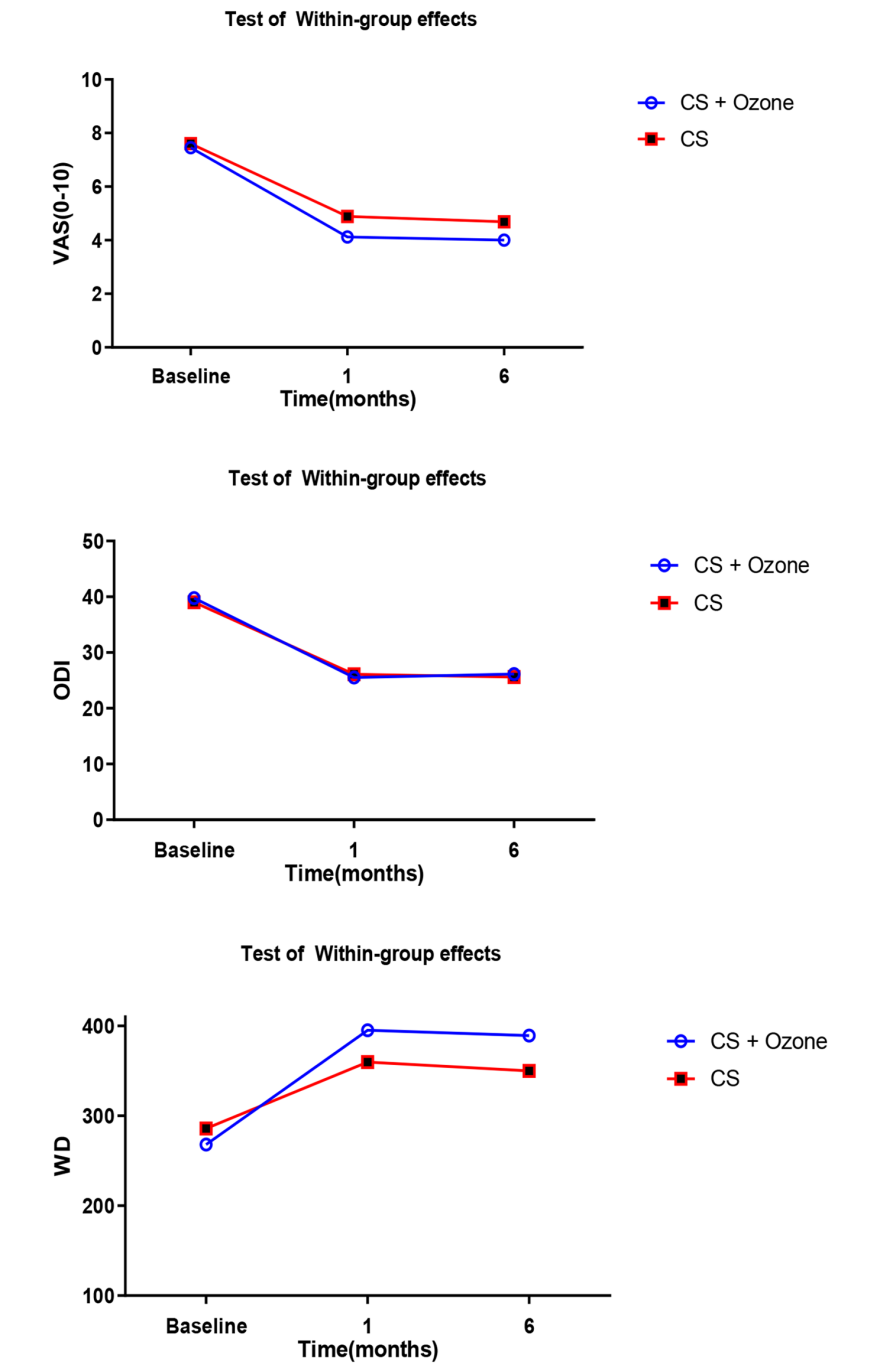
Comparison of changes in VAS, ODI and WD of patients with lumbar stenosis within the groups over time

In this study, the mean scores of ODI, WD and VAS in the two groups were examined in three time periods before the intervention, one month and six months after the intervention as the within-group differences; the results are reported in Table 2; Fig. 2. Based on these results, the mean differences in ODI, WD and VAS scores significantly improved one month and six months after treatment compared to before the intervention in both groups (P < 0.001). In this study, the mean ODI, WD and VAS scores in the two groups in the three time periods before the intervention, one month and six months after the intervention were examined as the between-group differences and the results are summarized in Table 2; Fig. 2.

**Table 2:** Comparison of changes in VAS, ODI and WD of patients with lumbar stenosis within and between the groups over time

Var	Time	Test of Within-group effects (Mean change from baseline)				Test of between-group effects (Mean change from group)			
		CS	P-value ^a^	CS + Ozone	P-value ^b^	MD	95% CI	P-value ^c^	Effect size
VAS	T1	-2.73 (-3.51, -1.96)	< 0.001	-3.33 (-4.10, -2.55)	< 0.001	-0.59	(-1.69, 0.50)	0.289	0.29(0.23, 0.35)
	T6	-2.91 (-3.69, -2.13)	< 0.001	-3.45(-4.23, -2.68)	< 0.001	-0.54	(-1.64, 0.55)	0.330	0.27 (0.21 ,0.31)
ODI	T1	-14.26 (-16.76, -11.75)	< 0.001	-12.87 (-15.79, -9.95	< 0.001	1.38	(-2.47, 5.24)	0.482	0.19(-0.37, 0.76)
	T6	-13.65 (-16.15, -11.14)	< 0.001	-13.37 (-16.29, -10.45)	< 0.001	0.27	(-3.58, 4.13)	0.88	0.03 (-0.54 ,0.60)
WD	T1	73.91(42.22,105.59)	< 0.001	127.08 (92.96,161.2)	< 0.001	53.17	(6.51,99.82)	0.026	0.57(0.01 1.15)
	T6	64.13(32.44 ,95.81)	< 0.001	121.04 (86.92,155.15)	< 0.001	56.91	(10.25,103.56)	0.017	0.62 (0.04 ,1.21)

**P-value**^**a**^: Adjusted generalized estimating equations model after controlling the baseline Outcome, sex, age, BMI, DM

**P-value**^**b**^: (|Baseline − 6th month|/Baseline) *100

**P-value**^**c**^: Adjusted generalized estimating equations model after controlling the baseline Outcome, sex, age, BMI, DM

The difference in the mean ODI and VAS scores was not statistically significant in the group receiving caudal epidural steroid injection + ozone compared to the group receiving epidural steroid injection alone in the first and sixth months (p > 0.05). However, the mean difference in WD score was 53.17 (6.51, 99.82) after one month, and this difference was statistically significant (p = 0.026), as well as 56.91 (10.25, 103.56) after six months and the difference was statistically significant (p = 0.017) in favor of the group receiving caudal epidural steroid injection + ozone.

## Discussion

According to this study, addition of ozone to triamcinolone does not have an added benefit in the treatment of pain in patients with lumbosacral canal stenosis. But it can be effective in some parts of patients’ performance, especially the walking ability. By comparing the ozone plus triamcinolone group with triamcinolone group and examining primary and secondary outcomes, there was no significant difference between VAS and ODI in the two groups in various time points. The only significant difference was the superiority of ozone plus triamcinolone over triamcinolone in WD outcome one month and six months post injection.

A 2004 study by Muto et al. Published fascinating results on ozone injection in 2,200 patients. In this study, intradiscal and intraforaminal/epidural ozone injections were used to assess the response to treatment of patients with lumbar disc herniation. According to this large study, ozone can cause good to excellent results in 75% of patients followed up for 18 months. It was also associated with a reduction in herniation size in 63% of patients. In addition to favorable therapeutic outcomes, no serious neurological or infectious complications have been reported in this study. The beneficial effects of ozone in this study have been linked to (1) improved oxygenation and reduced inflammation, (2) direct effects of ozone on mucopolysaccharides in nucleus pulposus and disc shrinkage, and finally, (3) improved microcirculation by reducing venous stasis [12]. The trial of Muto et al. may help us in explaining ozone injection’s pathophysiology and clinical results. In the Muto et al. study, intraforaminal injection was utilized simultaneously with intradiscal injection. Although, the results of the Muto study cannot only be attributed to epidural injections because of intradiscal procedures. The mechanism of improving microcirculation and reducing vascular stasis may explain the better results of the ozone group in the walking distance outcomes in our study. However, the anti-inflammatory effects of ozone may also play a role in improving the function of nerve roots and reducing radicular symptoms.

Among the few other studies published on epidural ozone injection is the Ryska trial in 2021. In this study, three types of treatments in lumbosacral radicular pain were carried out: transforaminal epidural steroid injection, transforaminal epidural ozone injection, and pulse radiofrequency of DRG. Assessment of VAS and ODI was done immediately after treatment, 3 months and 6 months later. The best response immediately after treatment was in the transforaminal steroid injection group, but there was no significant difference between the groups at 3 months and 6 months. Two cases of transient mild side effects, including nausea and headache after ozone injection, have been reported [18]. The only similarity between this study and our study is the use of ozone gas epidurally, which in this study was transforaminal and in our study was caudal. Ozone in the Ryska study, alone and without corticosteroids, has produced good outcomes within 6 months. Ozone in combination with steroids in our study could not boost the effects of corticosteroids except in WD outcomes. In Ryska study, no serious side effects were seen with epidural ozone injection.

Barbosa et al. performed epiduroscopy with concomitant ozone injections in patients with FBSS. Patients with ozone injection showed pain reduction within 21 days of follow-up in various outcome measures, including VAS, Brief Pain Inventory, Neuropathic Pain Symptom Inventory and Douleur Neuropathique 4. At the same time, there was no evidence of improved functional scales. The hypothesis for reducing pain in these patients is scar tissue adhesiolysis causing pain reduction and dehydration of the herniated discs. In addition, reducing the inflammatory processes through various cytokines is another possible cause of relieving pain [19]. Caudal epidural injection of ozone is similar to our trial. However, the dose and volume of ozone used in this study are 30 ug/mL and 20 mL, respectively, which is higher than ours and there is no combination with corticosteroid and anesthetic.

Several studies have scrutinized the influence of adding corticosteroids to ozone in treating radiculopathy with intradiscal and/or transforaminal approaches. The results of ozone and steroid combination in most studies favor the cumulative effects of ozone alongside steroids. The use of a combination of ozone and corticosteroids does not reduce the therapeutic effects and can cause additive effects in treating radiculopathy. The results of our study are in the same direction [20–24].

In general, the results of using ozone in treating lumbar problems and radiculopathy caused by disc herniation have been reported positively. Ozone, whether intradiscal or epidural or paravertebral, can improve the patient’s symptoms and is a practical step before surgical recommendation [25]. These positive therapeutic effects can last up to 10 years after the injection of ozone intradiscally [26].

Manchicanti et al. proposed that lidocaine alone or combined with steroids in epidural injections was beneficial in lumbosacral stenosis and radiculopathy. However, steroid itself, in combination with sodium chloride or bupivacaine, was not effective in improving symptoms. Despite numerous trials mentioning the usefulness of steroids, a body of literature question its effects [27, 28]. The definitive answer to whether the beneficial effects of caudal injections are due to anesthetics, corticosteroids, sodium chloride, or distilled water is not the purpose of this study. Still, at the same time, the answer may not be easy. Therefore, although the trial title mentions the comparison of steroid injection with or without ozone, it may actually be a comparison between bupivacaine with the combination of bupivacaine and ozone.

Ozone injection studies in lumbar disc herniation and radiculopathy are mostly intradiscal or a combination of intradiscal and epidural approaches [20, 29–31]. There is little research on using ozone alone or in combination with steroids and other treatments in epidural injections, such as the caudal approach. There is also no specific guideline for determining the best effective dose and number of epidural ozone injections.

We have no idea that the improvement in the walking distance in the ozone group is due to the beneficial effects of ozone including anti-inflammatory properties [32] or due to its mechanical pressure that helps bupivacaine and triamcinolone to reach higher levels in spinal column.

In our study, two cases of headache were reported in the corticosteroid plus ozone group, but there was no headache in the steroid group. A case of temporary urinary incontinence happened in the corticosteroid group, which recovered rapidly. Overall, the side effects were small and limited. In most of the studies, the side effects of ozone were small and temporary [12, 13, 18]. The points raised in Vanni’s study about root adhesion to dura and disc in intraforaminal ozone injections should be considered in future studies in caudal epidural ozone injection [33].

The use of ultrasound and color Doppler greatly increases the accuracy of caudal injection, and even in some studies, this accuracy has been reported up to 100% compared to fluoroscopy, in our opinion, the lack of fluoroscopy to confirm the proper injection site is one of the disadvantages of this study [34]. In rare cases, the intravascular injection may have been performed despite the observation of turbulence and lack of blood aspiration.

Research limitations include the small number of patients recruited and the short follow-up period, lack of consensus on the dose and volume of injectable ozone, and the number of injections. It is not yet clear whether multiple injections can have higher efficacy. Perhaps it would have been better to use ozone without triamcinolone and bupivacaine in another group to confirm the inherent benefits of ozone injection but this was not morally right because we were depriving the patient of the benefits of more proven treatments. Novel group design is the main advantage of this trial. To date, no study has examined the benefits of ozone in addition to triamcinolone and bupivacaine in caudal epidural injections. Using both subjective and objective outcome measures is the other advantage of this research.

## Conclusion

In this study, the results of VAS and ODI outcomes showed that caudal epidural steroid injection with ozone does not have significant advantage over caudal epidural steroid injection alone. Interestingly, our results demonstrated that the group receiving caudal epidural steroid injection with ozone had a significantly higher score of walking distance than the group receiving caudal epidural steroid. The results of this study raise the possibility that ozone alone or in combination with steroids and bupivacaine may help improve patients’ performance. Therefore, there is a need for further studies with a larger sample size in patients with spinal stenosis.

## Data Availability

The datasets used and/or analyzed during the current study available from the corresponding author on reasonable request.

## List of abbreviations

LBP: Low back pain
FBSS: Failed back surgery syndrome
BMI: Body mass index
MRI: Magnetic resonance imaging
VAS: Visual Analog Scale
ODI: Oswestry Disability Index
WD: Walking Distance
